# Ferroptosis is a new therapeutic target for spinal cord injury

**DOI:** 10.3389/fnins.2023.1136143

**Published:** 2023-03-14

**Authors:** Xin-Yue Bai, Xiao-Long Liu, Zhi-Zhong Deng, Dong-Min Wei, Die Zhang, Hui-Lin Xi, Qing-Yan Wang, Meng-Ze He, Yan-Ling Yang

**Affiliations:** School of Medicine, Yan’an University, Yan’an, China

**Keywords:** spinal cord injury, Ferroptosis, Ferroptosis inhibitors, clinical translation, animal models

## Abstract

Spinal cord injury is a serious traumatic disease. As Ferroptosis has been increasingly studied in recent years, it has been found to be closely related to the pathophysiological processes of spinal cord injury. Iron overload, reactive oxygen species accumulation, lipid peroxidation and glutamate accumulation associated with Ferroptosis are all present in spinal cord injury, and thus Ferroptosis is thought to be involved in the pathological processes secondary to spinal cord injury. This article highlights the relationship between Ferroptosis and spinal cord injury, lists substances that improve spinal cord injury by inhibiting Ferroptosis, and concludes with a discussion of the problems that may be encountered in the clinical translation of Ferroptosis inhibitors as a means of enabling their faster use in clinical treatment.

## 1. Introduction

Spinal cord injury (SCI) is a serious traumatic condition (Quadri et al., 2020) that is most commonly caused by car accidents and falls (Zipser et al., 2022). The global incidence rate is 10.4–83 cases per million per year (Karsy and Hawryluk, 2019), and SCI is associated with a high rate of disability and mortality (Chen C. et al., 2022), placing a severe physical burden on the patient. In addition, SCI is associated with a higher incidence of psychological disorders such as depression and anxiety than the general population (Hearn and Cross, 2020). The annual cost of treatment and care for each SCI patient has been reported to be as high as US$77,334 (Lo et al., 2021). However, in current clinical management, the treatment of SCI mainly includes surgery and related anti-inflammatory and anti-swelling medication (Liu X. et al., 2021). However, due to the complex pathophysiological process of SCI, the current treatment outcome is not satisfactory. Therefore, it is particularly important to explore the pathological process of SCI and new treatment methods.

Ferroptosis is a type of programmed cell death (PCD) (Shi et al., 2021). Its main mechanism is to catalyze the lipid peroxidation of polyunsaturated fatty acids (PUFA) highly expressed on cell membranes in the presence of divalent iron (Fe^2+^) or lipoxygenase (LOX), thereby inducing cell death (Ursini and Maiorino, 2020). It is mainly characterized by a decrease in the core enzyme glutathione peroxidase 4 (GPX4), the regulatory core of the antioxidant system glutathione (GSH) system (Wu et al., 2021). In recent years, Ferroptosis has been extensively studied in a number of conditions including cardiovascular disease (Wu et al., 2021), renal disease (Ni et al., 2022), and neurological disorders (Song and Long, 2020).

The pathological process of SCI is divided into primary and secondary injury (Shen and Cai, 2023), with primary injury being irreversible and secondary injury being reversible (Liu X. et al., 2021). Secondary injury is a cascade amplification response triggered by primary injury, which is divided into three stages: acute, subacute, and chronic injury, manifested as post-traumatic inflammatory response, free radical formation, and PCD of cells (Alcántar-Garibay et al., 2022). While PCD includes autophagy, necroptosis, cell scorching, and Ferroptosis, among others (Li et al., 2022), and it has been recently demonstrated that iron overload (Gong et al., 2022), ROS accumulation (Feng et al., 2021), lipid peroxidation (Baazm et al., 2021), and glutamate accumulation (Zhao et al., 2022) associated with Ferroptosis are all present in SCI, it is therefore suggested that Ferroptosis may be involved in the pathological process of secondary injury associated with SCI.

There is a large body of literature that demonstrates the close association of Ferroptosis with SCI, but very little literature that specifies the involvement of Ferroptosis in the pathological process of SCI. There is also little literature summarizing the substances that repair SCI by inhibiting Ferroptosis. And there is no literature that identifies the problems that may arise in the clinical translation of Ferroptosis inhibitors associated with SCI. This review therefore first describes the basic process of Ferroptosis and its close relationship with SCI, then briefly outlines the substances currently available to ameliorate SCI by inhibiting Ferroptosis, thus demonstrating that Ferroptosis is involved in the pathophysiology of SCI and that we can repair SCI by inhibiting Ferroptosis. Finally, this review discusses the problems that may be encountered in the clinical translation of Ferroptosis inhibitors associated with SCI and looks at the future direction of Ferroptosis research in SCI. It opens up a new way of thinking for the treatment of SCI.

## 2. The basic process of Ferroptosis and its relation to SCI

### 2.1. Iron metabolism

**FIGURE 1:**
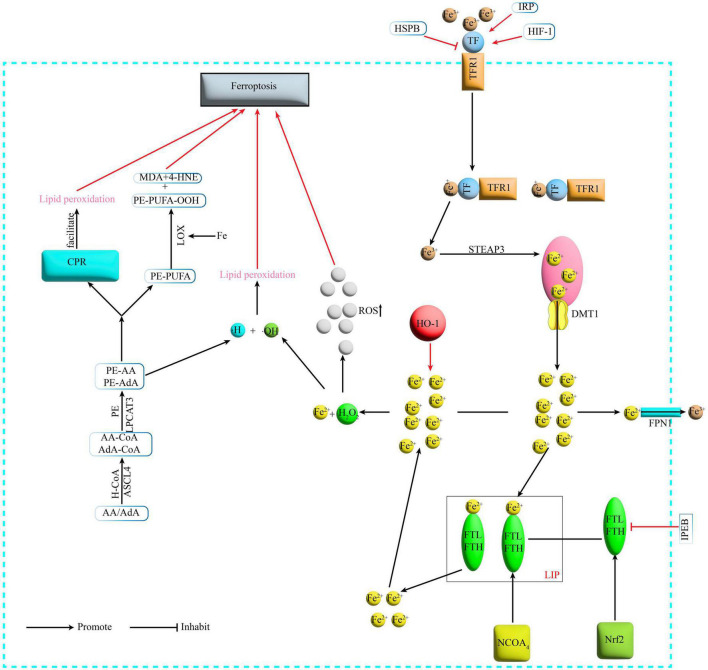
Mechanism of Ferroptosis. Fe^3+^ binds to TF on the cell membrane and is transported via TFR1 on the cell membrane surface, and the TFR1-TF-Fe^3+^ complex enters the cell by endocytosis. Under acidic conditions, Fe^3+^ is released from the binding and reduced by STEAP3 to Fe^2+^, which then enters the cytoplasm via DMT1 from the endosome. In the cytoplasm Fe^2+^ is partly transported outside the cell and converted to Fe^3+^ by FPN1, partly bound to ferritin to form LIP, and NCOA4 can recognize and rely on the autophagic pathway to degrade ferritin in the iron pool, releasing large amounts of Fe^2+^. TF, transferrin; TFR1, transferrin receptor1; STEAP3, six-transmembrane epithelial antigen of prostate 3; DMT1, divalent metal transporter 1; FPN1, membrane iron transporter 1; FPN1, ferroportin1; FTL, ferritin light chain; FTH, ferritin heavy chain; LIP, labile iron pool; NCOA4, nuclear receptor coactivator 4; H_2_O_2_, hydrogen peroxide; ACSL4, acyl-CoA synthetase long-chain family member 4; LPCAT3, lyso-phosphatidylcholine acyltransferase-3; LOX, lipoxygenase; MDA, malondialdehyde; 4-HNE, 4-hydroxynonenal; POR, cytochrome P450 oxidoreductase; IREB2, iron-responsive element-binding protein 2; HSPB1, heat shock protein beta-1; IRP, iron- regulatory protein; HIF-1, hypoxia-inducible factor-1; HO-1, heme oxygenase-1.

Iron is one of the important trace elements essential to the human body (Das et al., 2020). Abnormal iron metabolism is an important cause of Ferroptosis. When intracellular iron is overloaded, on the one hand, large amounts of Fe^2+^undergo Fenton reaction with hydrogen peroxide (H_2_O_2_), generating hydroxyl radicals with stronger oxidative capacity, thus increasing intracellular levels of reactive oxygen species (ROS) and promoting lipid peroxidation and ultimately Ferroptosis (Liu J. et al., 2022). On the other hand, Fe is a cofactor that enhances the activity of various metabolic enzymes such as LOX, which directly catalyze lipid peroxidation and induce Ferroptosis (Tang D. et al., 2021; Figure 1).

Basic experiments have found that in animal models of SCI, there is massive erythrocyte rupture at the site of injury, which can be observed as early as 1 h after injury and more commonly at 24 h (Wang Z. et al., 2022). In contrast, massive bleeding leads to increased iron concentrations at the site of injury (Gong et al., 2022), followed by Ferroptosis from ROS accumulation (Feng et al., 2021). Other researchers conducted *in vivo* and *in vitro* experiments with SCI rats and primary cortical neurons and found that there was significant microglia activation in the motor cortex after SCI resulting in the release of excess nitric oxide (NO), and that the large amount of NO interfered with the expression of proteins related to iron metabolism, ultimately leading to iron overload in the motor cortex of SCI rats at 4 weeks, resulting in ROS accumulation leading to neuronal Ferroptosis (Feng et al., 2021). Furthermore, the researchers found that iron deposition in the motor cortex was significantly increased in SCI patients compared to healthy controls. This triggered the accumulation of ROS, lipid peroxidation, mitochondrial atrophy and dysregulation of genes related to Ferroptosis, which ultimately led to Ferroptosis in motor neurons (Feng et al., 2021). And there are also many neurological disorders such as stroke (Cao et al., 2018), traumatic brain injury and neurodegenerative diseases (Daglas and Adlard, 2018) whose pathogenesis is linked to iron overload in the brain.

### 2.2. Lipid metabolism

Studies have shown that the polyunsaturated fatty acid (PUFA) family of arachidonic acid (AA)/adrenaline (AdA) undergoes acylation and lipidation to phosphatidylethanolamine-polyunsaturated fatty acids [Phosphatidyl Ethanolamine (PE-PUFA)] (Kagan et al., 2017). Ultimately PE-PUFA is involved in the downstream process of Ferroptosis, which can be divided into two pathways: (i) non-enzymatic oxidation (Conrad and Pratt, 2019) and (ii) enzymatic oxidation. Non-enzymatic reactions involve the Fenton reaction involving Fe^2+^ to produce hydroxyl radicals (HO-) that deprive lipids of H- to form lipid radicals, which then form lipid peroxides, leading to lipid peroxidation leading to Ferroptosis (Conrad and Pratt, 2019). In contrast, two pathways have been identified for enzymatic oxidation reactions. One is the formation of oxides PE-PUFA-OOH and related derivatives such as malondialdehyde (MDA) and 4-hydroxynonenal (4-HNE), which in turn react with DNA bases, proteins and other nucleophilic molecules, catalyzed by LOX leading to severe cytotoxicity (Kagan et al., 2020). Another pathway is the promotion of lipid peroxidation by cytochrome P450 oxidoreductase (POR), which leads to Ferroptosis (Zou et al., 2020; Figure 1).

It has been shown that the spinal cord contains high levels of polyunsaturated fatty acids, which have been shown to be involved in the oxidative stress response following SCI (Baazm et al., 2021). Studies on the regulation of SCI by unsaturated fatty acids such as short-chain fatty acids (SCFA) have been widely reported in recent years (Filippone et al., 2020). Thus suggesting that lipid metabolism in Ferroptosis may be involved in the pathological process of SCI. And SCFAs are also major metabolites produced by fermentation of dietary fiber bacteria in the gastrointestinal tract. It has been shown that SCFAs can directly or indirectly affect the brain-gut axis and have a mediating role in the microbiota-gut-brain axis (Dalile et al., 2019). Moreover, SCFAS is also a metabolite of intestinal bacteria and its concentration depends on the composition of the intestinal microbial population (Markowiak-Kopeć and Śliżewska, 2020). In contrast, Ferroptosis is an emerging mode of cell death in which lipid metabolism plays a crucial role (Conrad and Pratt, 2019). We therefore speculate that Ferroptosis, intestinal flora and the brain-gut axis may be linked around lipid metabolism as a regulatory factor, which may also provide some ideas and directions for future studies.

### 2.3. System Xc-GSH-GPX4 axis

**FIGURE 2:**
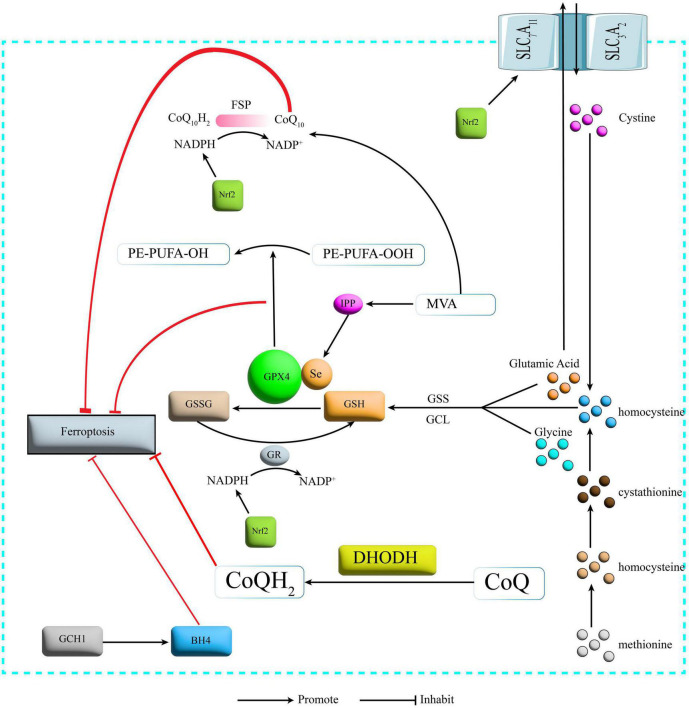
Protection mechanism for Ferroptosis. SLC7A11, recombinant solute carrier family 7, member 11; SLC3A2, recombinant solute carrier family 3, member 2; GSS, glutathione synthetase; GCL, glutamate cysteine ligase; GSH, glutathione; GPX4, glutathione peroxidase 4; GR, glutathione reductase; GSSG, glutathione oxidized; NADPH: nicotinamide adenine dinucleotide phosphate; NADP, nicotinamide adenine dinucleotide phosphate; FSP1, Ferroptosis inhibitory protein 1; CoQ10, Coenzyme Q10; DHODH, dihydroorotate dehydrogenase; CoQ Coenzyme Q; GCH1, GTP cyclohydrolase-1; BH4, tetrahydrobiopterin; IPP, isopentenyl pyrophosphate; MVA, mevalonate; Nrf2, nuclear factor erythroid 2-related factor 2.

The cystine/glutamate antiporter (System Xc-) transports glutamate out of the cell and transports cystine into the cell, where the transported cystine is reduced to cysteine (Parker et al., 2021) and then combined with glutamate and glycine in the presence of glutathione synthetase (Wu et al., 2022) (GPX4 is a selenocysteine-containing and GSH-dependent enzyme that is a key regulator of Ferroptosis (Seibt et al., 2019). It converts GSH to oxidized glutathione (GSSG) and reduces lipid peroxidation, thereby reducing lipid peroxide formation and oxidative stress damage and ultimately inhibiting Ferroptosis (Forcina and Dixon, 2019; Figure 2). Currently modulating cystine uptake, interfering with GSH and the expression of GPX4 remain the most common means of Ferroptosis in basic research.

One study found that GPX4 knockout-induced degeneration of spinal motor neurons exhibits Ferroptosis, and when supplemented with the Ferroptosis inhibitor vitamin E delayed the onset of paralysis and death in GPX4 knockout-induced mice (Chen et al., 2015). And some researchers have observed downregulation of GPX4 and upregulation of acyl-CoA synthetase long-chain family member 4 (ACSL4) in the acute phase of an animal model of SCI (Zhou et al., 2020). It can therefore be hypothesized that the pathological process of SCI is closely related to the Xc-GAH-GPX4 axis, a protective mechanism system for Ferroptosis.

### 2.4. NADPH-FSP1-CoQ10 pathway

CoQ10 is a lipophilic free radical trapping antioxidant that blocks the delivery of lipid peroxides and thus inhibits Ferroptosis (Liu J. et al., 2021). The glutathione non-dependent Ferroptosis inhibitory protein (Ferroptosis inhibitory protein 1, FSP1) is one of the redox enzymes of coenzyme Q10 (CoQ10), and FSP1 can, in the presence of reduced coenzyme II (nicotinamide adenine dinucleotide phosphate, NADPH) to reduce CoQ10 to reduced coenzyme Q10 (CoQ10H2) and also catalyze the regeneration of CoQ10 via NAD(P)H (Santoro, 2020; Figure 2).

It has been found that in animal models of SCI, the IncGm36569/miRNA-5627-5p/FSP1 axis was inhibited by molecular sponge action and thus targeting of the IncGm36569/miRNA-5627-5p/FSP1 axis to inhibit neuronal Ferroptosis (Shao et al., 2022). In the NADPH-FSP1-CoQ10 pathway, FSP1 has already been reported in basic studies at the molecular level as another target protein for Ferroptosis. Future studies on FSP1 may not only target the molecular level, but also natural and synthetic drugs that interfere with FSP1 expression may be one of the hot spots for future Ferroptosis studies.

### 2.5. DHODH-CoQH2 pathway

The dihydroorotate dehydrogenase-dihydroubiquione (DHODH-CoQH2) pathway is the third major Ferroptosis protection system on mitochondria in addition to the GPX4 pathway on the cytoplasm and mitochondria and the FSP1 pathway on the plasma membrane (Mao et al., 2021). CoQH2 acts as an antioxidant to eliminate lipid peroxyl radicals and thus inhibit Ferroptosis (Mao et al., 2021; Figure 2). Little linkage of SCI to this pathway has been reported and future studies could focus on this pathway.

### 2.6. GCH1-BH4 pathway

The GTP cyclohydrolase-1-tetrahydrobiopterin (GCH1-BH4) pathway is a lipid antioxidant pathway independent of the Xc-GSH-GPX4 axis and the NADPH-FSP1-CoQ10 pathway (Liu M. et al., 2022). GTP cyclohydrolase-1 (GTP Cyclohydrolase-1, GCH1) is a rate-limiting enzyme of BH4 (Larbalestier et al., 2022). overexpression of GCH1 enhances the production of BH4 (Kraft et al., 2020), a potent free radical-trapping antioxidant that protects cells from Ferroptosis by reducing lipid peroxidation (Vasquez-Vivar et al., 2022; Figure 2).

It has been shown that the GCH1-BH4 metabolic pathway is a protective mechanism for cellular Ferroptosis and that GCH1 inhibitors are a novel therapeutic measure for the treatment of colorectal cancer (Hu et al., 2022). However, this pathway has been largely unreported in SCI, and the development of related studies will certainly enhance the emerging field of Ferroptosis.

### 2.7. The MVA pathway

The mevalonate (MVA) pathway plays a key role in the regulation of Ferroptosis (Yao et al., 2021). the MVA pathway can influence GPX4 synthesis by regulating the maturation of selenocysteine tRNA (Warner et al., 2000), while CoQ10 can be synthesized using acetyl-coenzyme A (Acetyl-CoA) via the MVA pathway (Ball et al., 2021). The MVA pathway therefore links the Xc-GSH-GPX4 axis and the NADPH-FSP1-CoQ10 pathway (Figure 2). However, little research has been reported on the inhibition of Ferroptosis and thus repair of SCI through modulation of the MVA pathway. In contrast, MVA, as a synthetic precursor of cholesterol (Guerra et al., 2021), also has a considerable role in lipid metabolism. The MVA pathway therefore re-emphasizes the role of lipid metabolism in Ferroptosis. Aerobic oxidation, glucose metabolism and lipid metabolism play a crucial role in life activities, all three being interconnected and independent of each other. The role of lipid metabolism in Ferroptosis has been widely reported (Baazm et al., 2021). In contrast, the role of aerobic oxidation and sugar metabolism in Ferroptosis has hardly been reported. Some researchers have found that the role of both the mitochondrial tricarboxylic acid cycle (TCA cycle) and the electron transport chain (ETC) is required for Ferroptosis, and that the mitochondrial ETC can regulate Ferroptosis induced by cysteine deprivation In contrast, the mitochondrial TCA cycle can be involved in cysteine deprivation-induced Ferroptosis (Gao et al., 2019). In turn, the tricarboxylic acid cycle is the ultimate metabolic pathway for the three major nutrients (sugars, lipids, and amino acids) and is the hub for the metabolic linkage of sugars, lipids, and amino acids (Martínez-Reyes and Chandel, 2020). We therefore hypothesize that aerobic oxidation as well as sugar metabolism may also have an integral role in Ferroptosis. Future studies that focus on this will be of great significance for Ferroptosis and related areas of research.

### 2.8. Nrf2

Nuclear factor erythroid 2-related factor 2 (Nrf2) is an important anti-oxidative stress transcription factor that improves cellular tolerance to oxidative stress (He et al., 2020). It has been shown that its downstream target proteins and enzymes include (i) proteins related to iron metabolism: ferritin light chain (FTL), ferritin heavy chain (FTH), and ferroportin1 (FPN1), which is responsible for iron transport (Chen et al., 2021). (ii) Enzymes related to NADPH regeneration: glucose-6-phosphate dehydrogenase (G-6-PD), phosphogluconate dehydrogenase (PGD), and malic enzyme (ME) (Zhang H. S. et al., 2019). ME) (Zhang H. S. et al., 2019), and NADPH regeneration is essential for GPX4 activity. (iii) Proteins and enzymes related to GSH metabolism: Solute Carrier Family 7 Member 11 (SLC7A11), a subunit of the cystine/glutamate transporter protein xCT (Dong et al., 2020) and GSS (Zheng et al., 2014). All these proteins and enzymes are involved in the regulation of Ferroptosis and therefore Nrf2 is considered a key regulator of Ferroptosis (Dodson et al., 2019) and has been a star target in Ferroptosis studies in recent years (Figure 2).

In summary, the two most critical targets for the inhibition of Ferroptosis initiation, xCT and GPX4, have been modulated by NRF2. It has also been shown that Nrf2 can be used to regulate Ferroptosis to treat neurodegenerative diseases (Song and Long, 2020), delay the progression of diabetic nephropathy (DN) (Li S. et al., 2021) and prevent acute lung injury due to intestinal ischemia/reperfusion (Dong et al., 2020), and it has been found that proanthocyanidins (Zhou et al., 2020), alginose (Gong et al., 2022), metformin (Wang H. et al., 2020), and growth differentiation factor-15 (Xia et al., 2022) are all inhibited by NRF2. These materials have been found to inhibit Ferroptosis through the Nrf2 pathway to treat SCI, but the specific downstream target proteins and enzymes that these substances affect through the Nrf2 pathway to inhibit Ferroptosis to treat SCI have not been clearly investigated. The above-mentioned roles of Nrf2 in various diseases demonstrate the importance of the Nrf2 pathway in Ferroptosis, which suggests that we need to focus more on the star targets such as Nrf2 in future Ferroptosis research, especially in the study of novel drugs, which may play an indispensable role in the application of Ferroptosis-related drugs and the treatment of related diseases.

## 3. Related substances that improve SCI by inhibiting Ferroptosis

### 3.1. Natural materials

#### 3.1.1. Proanthocyanidins

Proanthocyanidins (PACs), often extracted from grape seeds, are potent free radical scavengers (Wang C. et al., 2022). PACs have been shown to promote recovery of motor function in rats with SCI (Liu W. Z. et al., 2022). And in 2020 an investigator found that proanthocyanidins promote recovery of motor function in SCI mice by inhibiting Ferroptosis (Zhou et al., 2020). PACs inhibit the recovery of motor function in SCI mice by upregulating the expression of GSH, GPX4, Nrf2, and HO-1 and downregulating the expression of iron, ACSL4 and thiobarbituric acid reactive substances (TBARS). TBARS expression to suppress Ferroptosis in the microenvironment at the site of injury (Zhou et al., 2020). However, PACs treatment had no significant effect on the expression of Recombinant Lysophosphatidylcholine Acyltransferase 3 (LPCAT3) levels in SCI, so it is uncertain whether PACs inhibit Ferroptosis by affecting AA/AdA levels and further studies are needed. further study.

#### 3.1.2. Epigallocatechin gallate

Epigallocatechin gallate (EGCG) is a catechin isolated from green tea. It has very strong antioxidant activity, at least 100 times more active than vitamin C (Tang G. et al., 2020). EGCG has been shown to have neuroprotective effects in SCI (Wang F. et al., 2022). Zhang H. S. et al. (2019) researchers found that EGCG can upregulate the expression of GPX4 and FTH1 and downregulate the expression of ACSL4, thereby inhibiting Ferroptosis to promote recovery of motor function in rats after spinal cord transection (ST). Thus, EGCG may have a very good future as a promising novel natural substance in the clinical treatment of SCI.

#### 3.1.3. Carnosic

Carnosic acid is a natural phenolic diterpene that can be extracted from rosemary (Satoh et al., 2022). Cheng et al. (2021) showed that Carnosic acid could down-regulate the expression of iron, ROS and MDA and up-regulate the expression of GSH in Erastin-treated PC12 cells. It was further demonstrated that syringic acid inhibited Erastin-induced Ferroptosis in PC12 cells by activating the Nrf2 pathway (Cheng et al., 2021), further demonstrating that the Nrf2 pathway is closely related to Ferroptosis.

#### 3.1.4. Trehalose

Trehalose, a non-reducing disaccharide composed of two glucose molecules, is a typical stress metabolite (Zhang and DeBosch, 2019). Gong et al. (2022) first demonstrated that trehalose inhibited Ferroptosis of neurons after SCI in mice by activating the Nrf2/HO-1 pathway, thereby promoting neuronal survival and improving recovery of motor function. In addition, alglucan also inhibited the expansion of neural tissue cavities and suppressed neuronal loss and inflammatory responses in SCI mice (Gong et al., 2022).

### 3.2. Metabolites

#### 3.2.1. Lipoxin A4

Lipoxin A4 (LXA4) is a metabolite of arachidonate lipoxygenase (ALOXE). LXA4 has been previously shown to repair SCI by activating the Nrf2/HO-1 signaling pathway (Lu et al., 2018). Wei et al. (2021) used Erastin to induce Ferroptosis in neuronal cells, and LXA4 effectively alleviated the downregulation of GPX4, GSH, and cysteine, and prevented the downregulation of Prostaglandin Endoperoxide Synthase 2 (PTGS2), ACSL4, and ROS upregulation. This study provides strong evidence that LXA4 improves SCI even more.

#### 3.2.2. Colony-stimulating factor

Erythropoietin (EPO), also known as erythropoietin-stimulating factor, is a human endogenous protein hormone that stimulates erythropoiesis. EPO has been reported to improve recovery of motor function in rats with SCI (Zhong et al., 2020). However, whether its mechanism is related to Ferroptosis has not been clarified. Recently, some investigators found that EPO has similar effects to Ferrostatin-1, an Ferroptosis inhibitor, on inhibiting the expression of Ferroptosis-related proteins and restoring mitochondrial morphology. EPO was also found to increase the expression of xCT and GPX4. Thus suggesting a potential anti-Ferroptosis effect of EPO (Kang et al., 2023), further improving the rationale for the use of EPO in the clinic.

### 3.3. Drug

#### 3.3.1. Edaravone

Edaravone is a free radical scavenger (Dang et al., 2022). Pang et al. (2022) found that edaravone upregulated GPX4/xCT and downregulated ACSL4/5-LOX to inhibit Ferroptosis during the acute phase of SCI in rats, thereby improving recovery of motor function in SCI.

#### 3.3.2. Metformin

Metformin (Met) is an organic compound that is a first-line drug for the treatment of type 2 diabetes (Flory and Lipska, 2019). One study found that Met promotes axonal regeneration after SCI via the Nrf2 pathway (Wang H. et al., 2020). And Met has also been found to upregulate GPX4 expression (Ma et al., 2021) and reduce MDA levels (Zeng et al., 2019), thereby improving recovery of motor function in rats with SCI (Wang Z. et al., 2022).

Taken together, these substances can improve motor recovery after SCI by inhibiting Ferroptosis, providing further evidence for their clinical application and also suggesting that SCI is closely related to Ferroptosis and that Ferroptosis will be a new target for the treatment of SCI in the future.

### 3.4. Trace elements

#### 3.4.1. Zinc

Zinc is an essential trace element in the human body (Kim and Lee, 2021). Researchers found that zinc increased the expression of Nrf2/HO-1 and thus upregulated GPX4 and GSH in a mouse model of SCI, and that zinc also effectively prevented oxidative stress in mitochondria and effectively reduced inflammation (Ge et al., 2021).

#### 3.4.2. Selenium

Selenium can replace sulfur in cysteine and is incorporated into selenoproteins as a selenosubstituted cysteine. GPX4 is the most important of the selenoproteins in human 25 (Zhang and Song, 2021). Therefore selenium is essential for GPX4. One researcher injected sodium selenite in a rat model of SCI and found that sodium selenite treatment downregulated iron levels and the expression of lipid peroxidation products MDA and 4-HNE, in addition to finding that sodium selenite inhibited Ferroptosis via the FSP1/GPX4 pathway thereby improving recovery of motor function in SCI rats (Chen Y. X. et al., 2022).

The role played by zinc and selenium in SCI provides a solid theoretical basis and dosing recommendations for the clinical use of micronutrients that can protect against neurological injury.

### 3.5. miRNAs

miRNA is a non-coding RNA of 21-25 amino acids in length, which can inhibit translation or induce target mRNA degradation at the post-transcriptional level by binding to the 3′-untranslated region (UTR) in messenger RNA (mRNA) (Saliminejad et al., 2019). One study found that miRNA-672-3p could inhibit Ferroptosis via the FSP1 pathway, thereby improving motor function in rats (Wang F. et al., 2022). Since miRNA is an RNA that does not encode a protein, one target protein may be regulated by multiple miRNAs, and one miRNA may also regulate multiple target proteins, so it may also be involved in the regulatory mechanisms related to Ferroptosis. However, little research has been reported on Ferroptosis and miRNAs. More importantly, miRNAs are only one type of non-coding RNAs, and studies on non-coding RNAs such as IncRNAs, CirRNAs, and co-regulatory networks between non-coding RNAs and Ferroptosis have also been rarely reported. Therefore, future studies on Ferroptosis could focus more on the molecular level, especially in non-coding RNAs, to provide more targets for Ferroptosis studies at the molecular level.

### 3.6. Mesenchymal stem cell transplantation

Mesenchymal stem cells (MSCs) and long non-coding RNA (lncRNA) are an important class of exosome contents. It has been found that exosomes from MSCs are dependent on a novel lncRNA, Gm36569, which inhibits Ferroptosis in neuronal cells by competing for the expression of miR-5627-5p and thereby enhancing the expression of FSP1 (Shao et al., 2022). This study links MSC transplantation, exosomes, Ferroptosis and SCI closely together, further promoting the development of MSC transplantation in SCI and providing theoretical support for the clinical application of MSC transplantation.

### 3.7. Cytokines

Growth-differentiation-factor-15 (GDF-15) is a cytokine that is a member of the transforming growth factorβ (TGF-β) superfamily, which is a stress response protein (Wang D. et al., 2021). One researcher examined the level of GDF-15 in SCI and found that the expression level of GDF-15 was significantly elevated in SCI and in neuronal Ferroptosis *in vitro*. And the knockdown of GDF-15 significantly exacerbated Ferroptosis. Subsequent researchers have found that GDF-15 inhibits Ferroptosis by activating the p62-Keap1-Nrf2 signaling pathway, thereby improving motor recovery in SCI (Xia et al., 2022). However, other modulatory effects of GDF-15 on Ferroptosis and neuroinflammation in the neurocloud after SCI remain uncertain, and therefore further studies on the modulatory effects of GDF-15 are needed in the future.

### 3.8. Newly synthesized substances

#### 3.8.1. SRS16-86

SRS16-86 is a newly synthesized small molecule inhibitor of Ferroptosis (Linkermann et al., 2014). One study found that SRS16-86 upregulated the levels of XCT, GSH and GPX4 in rat SCI and downregulated the lipid peroxidation product 4-hydroxynonenalal (4-HNE) thereby inhibiting Ferroptosis. In addition, SRS16-85 inhibited the inflammatory response and astrocyte proliferation after SCI. SRS16-85 increased neuronal survival and improved motor recovery from SCI in rats by inhibiting Ferroptosis (Zhang Y. et al., 2019).

#### 3.8.2. DFO

Deferoxamine (DFO) is an effective iron chelator that has been approved by the Food and Drug Administration (FDA) for the treatment of iron overload disorders (Li et al., 2019). It has been found that DFO promotes the repair of rat SCI by upregulating the levels of XCT, GSH, and GPX4 and inhibiting lipid reactive oxygen species in rat SCI, which in turn upregulates Ferroptosis related genes Acyl-CoA synthase family member 2 (ACSF2) and iron response element binding protein 2 (IREB2) ultimately inhibiting the Ferroptosis pathway (Yao et al., 2019). The application of DFO directly indicates its great promise in Ferroptosis. This may accelerate the clinical application of DFO in SCI.

#### 3.8.3. Liproxstatin-1

In 2021 a study found that Liproxstatin-1 was more effective than edaravone and DFO in rescuing oligodendrocytes. And Liproxstatin-1 was found to not only inhibit mitochondrial lipid peroxidation but also restore the expression of GSH, GPX4, and FSP1 (Fan et al., 2021).

#### 3.8.4. Ferrostatin-1

Ferrostatin-1 was shown to inhibit Ferroptosis in neurons, thereby improving functional recovery after TBI and SCI (Xie et al., 2019). Ge et al. (2022) found that Ferrostatin-1 could inhibit Ferroptosis in oligodendrocytes by reducing the accumulation of iron and ROS and downregulating the Ferroptosis-related genes IREB2 and PTGS2 (Ge et al., 2022).

The above studies provide a theoretical basis for the clinical use of these substances in the treatment of SCI. In particular, studies of natural compounds and related molecules have confirmed the potential of these substances in Ferroptosis and clinical treatment. It is also clear from the above table that both natural and newly synthesized substances inhibit Ferroptosis and thus repair SCI mainly by affecting xCT, GSH, GPX4, FSP1, and Nrf2 factors (Table 1). However, this does not mean that substances that inhibit Ferroptosis and thus repair SCI only do so by affecting the above pathways, but may also inhibit Ferroptosis and thus repair SCI by affecting the DHODH-CoQH2 pathway, the GCH1-BH4 pathway and the MVA pathway, etc. However, there is little research on these pathways, so the targets of future Ferroptosis inhibitors for repairing SCI and Therefore, future studies on the targets of Ferroptosis inhibitors for SCI repair and the mechanisms of action of natural products that can inhibit Ferroptosis and thereby repair SCI could focus on these pathways.

**TABLE 1:** Improvement of SCI-related substances through Ferroptosis.

Classification	Substance	Type	Mechanism
Natural products	PACs	Free radical scavengers Antioxidants	Upregulation of GSH, GPX4, Nrf2 (Zhou et al., 2020) Downregulation of ACSL4, Fe, TBARS (Zhou et al., 2020)
	EGCG	Antioxidants	Upgraded GPX4, FTH (Wang J. et al., 2020) Downward revision of ACSL4 (Wang J. et al., 2020)
	Carnosic	Anti-inflammatory Neuroprotective	Upregulation of GSH, Nrf2 (Cheng et al., 2021) Downregulation of iron content, ROS, MDA (Cheng et al., 2021)
	Trehalose	Prevents lipid peroxidation Inhibits inflammation	Upregulates Nrf2 (Gong et al., 2022)
Metabolic products	LXA4	Anti-inflammatory	Upregulation of GSH, GPX4, Nrf2 (Wei et al., 2021) Downregulation of ACSL4, PTGS2, ROS (Wei et al., 2021)
	EPO	Colony-stimulating factor	Up-regulation of xCT GPX4 (Kang et al., 2023)
Drugs	Edaravone	Free radical scavenger	Upregulated xCT GPX4 (Pang et al., 2022) Downregulation of ACSL4, 5-LOX (Pang et al., 2022)
	Met	Glucose-lowering drugs	Upregulated GPX4, Nrf2 (Ma et al., 2021) Downregulation of MDA (Zeng et al., 2019)
Trace elements	Zinc	Trace elements	Upregulation of GSH, GPX4, Nrf2 (Ge et al., 2021) Downregulation of ROS (Ge et al., 2021)
	Selenium	Trace elements	Upregulation of GPX4 FSP1 (Chen Y. X. et al., 2022) Downregulation of MDA 4-HNE (Ge et al., 2021)
miRNA	miRNA-672-3p	RNA	Upregulates FSP1 (Wang F. et al., 2022)
Stem cell transplantation	lncRNA-Gm36569	RNA	Upregulates FSP1 (Shao et al., 2022)
Cytokines	GDF-15	Neuroprotective factor	Upregulated Nrf2 (Xia et al., 2022)
New synthetic substances	SRS16-86	Small molecule Ferroptosis inhibitors	Upregulates xCT, GSH, GPX4 (Zhang Y. et al., 2019)
	DFO	Iron chelator	Upregulates xCT, GSH, GPX4 (Yao et al., 2019)
	Liproxstatin-1	Ferroptosis Inhibitors	Upregulates xCT, GPX4, FSP1 (Fan et al., 2021)
	Ferrostatin-1	Ferroptosis Inhibitors	Downregulation of Iron, ROS, IPEB2, PTGS2 (Ge et al., 2022)

## 4. Possible problems in the clinical translation of Ferroptosis inhibitors for the treatment of spinal cord injury

Recently, DFO has been approved by the US Food and Drug Administration (FDA) for the treatment of iron overload disorders (Li et al., 2019), for example to reduce systemic iron load in patients with thalassemia major and sickle cell (Farr and Xiong, 2021). However, DFO currently has some limitations for clinical translation in other diseases. For example, in Intracerebral Hemorrhage (ICH), studies have shown that high doses of DFO are dangerous in treating patients with ICH (Farr and Xiong, 2021). In SCI, it has been shown that DFO can improve SCI by promoting neovascularization in rats, but there are limitations to this study such as the uncertainty of the therapeutic effect of DFO in the chronic phase of SCI and the need to verify the therapeutic effect of DFO in SCI in high quality clinical studies, of which there are few (Tang G. et al., 2020). Most other Ferroptosis inhibitors have not yet been translated for clinical use and face many challenges.

### 4.1. Mode of administration

#### 4.1.1. Injection localized at the traumatized spinal cord

In basic research, some investigators microinjected Ferrostatin-1, an Ferroptosis inhibitor, into the dorsal 2 mm cephalad and 2 mm caudal aspects of the spinal cord in a rat model of SCI and inhibited the accumulation of iron and ROS, resulting in improved functional recovery after SCI (Ge et al., 2022). Other studies have injected sodium selenite spinal cord into a rat model of SCI and found downregulation of iron levels, MDA and 4-HNE expression levels and functional recovery after SCI (Chen Y. X. et al., 2022). Although this method is accurate in its localization (Falsafi et al., 2021), this localization of drug delivery at the traumatized spinal cord may be difficult to manipulate when applied clinically and may predispose patients to secondary injury (Table 2).

#### 4.1.2. Intraperitoneal injection

Some researchers injected DFO (Yao et al., 2019) and SRS16-86 (Zhang Y. et al., 2019) intraperitoneally into a rat model of SCI and found that the Xc-GSH-GPX4 axis was upregulated to inhibit Ferroptosis, thereby improving the functional recovery of SCI in rats. However, whether the drug crosses the blood–brain barrier (BBB) or blood–spinal cord barrier (BSCB) during intraperitoneal injection for clinical application still deserves further study (Table 2).

#### 4.1.3. Intranasal administration

Intranasal administration has been shown to bypass the BBB or BSCB and allow access to the central nervous system in animal models (Long et al., 2020), and it has been shown that intranasal administration of Liproxstatin-1 and Ferrostatin-1 treatment significantly improved infarct size in a mouse stroke model (MCAO ischemic stroke model) (Tuo et al., 2017), so the mode of administration may be clinically applicable in the treatment of spinal cord injury (Table 2).

#### 4.1.4. Intravenous injection

Intravenous injection is probably the most suitable mode of administration for emergency treatment in clinical practice because of its ease of handling, high volume of administration and low risk. However, the ability of this mode of administration to cross the blood-brain barrier during clinical application is still debatable (Table 2). One investigator injected the third generation Ferroptosis inhibitor SRS16-86 (Zhang Y. et al., 2019) intravenously into mice and collected cerebrospinal fluid, brain tissue fluid and serum samples. Both cerebrospinal fluid and brain tissue fluid were found to be below detection levels, indicating that intravenous SRS16-86 did not cross the BBB.

In the treatment of central nervous system diseases, the BBB limits the ability of 98% of small molecules and almost all large molecules to reach the lesion effectively. Therefore, it is extremely important that the mode of administration of Ferroptosis inhibitors crosses the BBB when used in clinical practice. There are several ways to increase the BBB, for example (i) carotid artery injection of high concentrations of mannitol can deliver drugs to the brain that are not permeable to the BBB. However, this method is not clinically applicable as it is complex and has a high risk of causing seizures, cerebral artery embolism, cerebral hemorrhage and cerebral edema (Wang W. et al., 2021). (ii) Use of vasoactive agents to increase BBB permeability throughout the brain (Gao et al., 2014). (iii) Use of cell-penetrating peptides, adenovirus-associated virus (AAV) that penetrates the BBB (Yao et al., 2022) and receptor-mediated transcytosis to increase brain transport (Terstappen et al., 2021). (iv) Localized increase in brain penetration by ultrasound stimulation of microvesicles (Rezai et al., 2020). (v) Molecularly targeted nanoparticles irradiated by picosecond pulsed laser to reversibly open the BBB and deliver drugs to brain tissue, specifically by synthesizing a gold nanoparticles (AuNPs) that are specifically targeted intravenously to tight junctions (TJs) on the BBB, followed by cranial picosecond laser stimulation to increase BBB permeability, a strategy that allows immunoglobulin and viral gene therapy vectors and drug-laden liposomes to enter the brain, and a process that is reversible and does not result in spontaneous vascular diastole or significant disruption of the structure of the neurovascular unit (Li X. et al., 2021). Although the above possible solutions for BBB have not been studied in BMSC, they may also provide relevant ideas and inspiration for the clinical use of Ferroptosis inhibitors in SCI.

**TABLE 2:** Advantages and disadvantages of drug delivery methods.

Method of administration	Advantages	Disadvantages
SCI site injection	Accurate positioning (Falsafi et al., 2021)	May be difficult to use in clinical practice and may cause secondary harm to patients
Intraperitoneal injection	Easy to absorb (Dou et al., 2013)	Improper handling can easily cause damage to organs
Intranasal drug delivery	Easy to pass BBB or BSCB (Long et al., 2020)	Improper handling can easily lead to upper respiratory tract infections
Intravenous injection	Easy handling, high dosingand, low risk	Difficult to pass BBB or BSCB (Zhang Y. et al., 2019)

### 4.2. Dosing

#### 4.2.1. Selection based on preliminary experiments

For example, when the researchers selected the dose to be administered for SRS16-86, five groups were modeled as sham group, SCI group, SCI + 5 mg/kg SRS16-86 group, SCI + 10 mg/kg SRS16-86 group and SCI + 15 mg/kg SRS16-86 group, and then tested motor recovery 2 weeks after SCI to select a dose of 15 mg/kg SRS16-86 (Zhang Y. et al., 2019).

#### 4.2.2. Selection based on experience

For example the dose of 100 mg/kg administered for DFO was selected by the investigators with slight modifications based on previous studies.

The dose administered in the basic study was determined in both of these ways, but in the clinical setting the dose needs to be studied further, taking into account the patient’s age, weight and dosing pattern. If too small a dose is administered, it may not be effective, and if too large a dose is administered, adverse effects and side effects may occur.

### 4.3. Time window for dosing

Some of the Ferroptosis inhibitors in basic studies were administered before SCI, for example DFO was administered to rats 30 min before injury and then injected once a day for 7 days after injury. However, this dosing time window is not achievable in clinical applications and therefore needs to be further explored.

## 5. Conclusion and outlook

In summary: (1) The existing studies suggest that Ferroptosis is closely related to SCI and that Ferroptosis may be a new target for SCI treatment. (2) Most of the current studies on Ferroptosis in SCI are at the animal stage, and future studies could be conducted on human cell lines or non-human primates to lay the foundation for clinical development of Ferroptosis inhibitors. (3) Although there is increasing evidence that Ferroptosis inhibitors can be used as a new generation of targets for the treatment of SCI, future clinical translation may face a number of problems such as poor *in vivo* solubility, short half-life, dose and mode of administration. (4) In addition to Ferroptosis, studies on the characteristics of different death modes, such as cuproptosis (Tsvetkov et al., 2022) and parthanatos (Wang and Ge, 2020), as well as studies on the linkage between these death modes, are still a hot topic for future research.

## Author contributions

X-YB, X-LL, and Z-ZD were responsible for writing and revising the text. X-YB played a major role in writing this article. X-LL was responsible for the preparation of figures and revisions of this article. Z-ZD was responsible for the post-writing and revisions of this article. D-MW and DZ were responsible for data collection. H-LX, Q-YW, and M-ZH were responsible for revising the article. Y-LY was responsible for conceptualizing, funding, and guiding the article. All authors contributed to the article and approved the submitted version.
